# Novel high-throughput screening method using quantitative PCR to determine the antimicrobial susceptibility of *Orientia tsutsugamushi* clinical isolates

**DOI:** 10.1093/jac/dky402

**Published:** 2018-10-06

**Authors:** Weerawat Phuklia, Phonepasith Panyanivong, Davanh Sengdetka, Piengchan Sonthayanon, Paul N Newton, Daniel H Paris, Nicholas P J Day, Sabine Dittrich

**Affiliations:** 1 Department of Molecular Tropical Medicine and Genetics, Faculty of Tropical Medicine, Mahidol University, Bangkok, Thailand; 2 Lao-Oxford-Mahosot Hospital-Wellcome Trust Research Unit (LOMWRU), Microbiology Laboratory, Mahosot Hospital, Vientiane, Lao PDR; 3 Mahidol-Oxford Tropical Medicine Research Unit, Faculty of Tropical Medicine, Mahidol University, Bangkok, Thailand; 4 Centre for Tropical Medicine and Global Health, Nuffield Department of Medicine, University of Oxford, Oxford, UK; 5 Swiss Tropical and Public Health Institute, Basel, Switzerland; 6 University of Basel, Basel, Switzerland; 7 FIND, Malaria & Fever Program, Geneva, Switzerland

## Abstract

**Objectives:**

To develop a method to enable the large-scale antimicrobial susceptibility screening of *Orientia tsutsugamushi* clinical isolates, using one timepoint and one concentration of antibiotics to considerably speed up the time to result.

**Methods:**

Growth, harvesting, multiplicity of infection (moi) and the day to determine the MICs were optimized using five *O. tsutsugamushi* reference strains [susceptible (Karp, Kato and Gilliam) and putatively resistant (AFC-3 and AFSC-4)], one clinical isolate (UT76) and one rodent isolate (TA763). Subsequently, the MICs of azithromycin, chloramphenicol and doxycycline for these strains and 51 clinical isolates including AFSC-7 were determined. An optimal concentration was calculated using the epidemiological cut-off value.

**Results:**

The conditions for *O. tsutsugamushi* infection, growth and harvesting were determined to be an moi of 100:1 and trypsinization with the peak growth on day 10. The resulting MICs were in line with previously published susceptibility data for all reference strains, except for Karp and AFSC-4, which showed azithromycin MICs of 0.0156 and 0.0313 mg/L, compared with 0.0078 and 0.0156 mg/L, respectively, in previous reports. The MIC of doxycycline for AFC-3 was 0.125 mg/L compared with >4 mg/L in earlier reports. The final single screening concentrations were identified as: azithromycin, 0.125 mg/L; chloramphenicol, 8 mg/L; and doxycycline, 1 mg/L.

**Conclusions:**

This simplified procedure facilitates the simultaneous screening of 48 isolates for actively monitoring potential resistance of this important fever pathogen, with an 8-fold throughput improvement over early methods. The data do not support the existence of doxycycline- and chloramphenicol-resistant scrub typhus.

## Introduction 

*Orientia tsutsugamushi* is an obligate intracellular Gram-negative bacterium, the causative agent of scrub typhus. The disease is a leading cause of treatable fever in Asia and Northern Australia, with high incidences reported in Thailand, Laos and India.^1^^,^^2^ Recent reports from further afield, especially in South America, suggest that this is a far more global pathogen and a possible cause of febrile illness beyond the textbook-described ‘tsutsugamushi triangle’.^3^^,^^4^ Scrub typhus is also a major cause of CNS infection in Asia.^5–7^

Scrub typhus is regarded as treatable with doxycycline, azithromycin and chloramphenicol.^8^ However, clinical and laboratory evidence for doxycycline resistance in scrub typhus was described in Chiangrai, Northern Thailand, in 1996.^9^ Given the large burden of disease and the significant mortality associated with it, these reports sparked significant concern in the region.^10^ With the possible emergence of resistant strains, treatment guidelines may need to be reconsidered.

In 2014 the WHO released a report highlighting the significant threat posed by antimicrobial resistance (AMR) and its consequences. It is estimated that ∼700 000 people die from drug-resistant pathogens per year. These numbers are predicted to rise, with the majority of resulting deaths likely to occur in low- and middle-income countries.^11^^,^^12^ To tackle the global spread of resistance it is important to monitor AMR patterns for a multitude of pathogens. Antibiotic susceptibility testing (AST) is conventionally conducted on isolates using disc diffusion, and Etest and broth microdilution to determine the MICs of antibiotics.^13^ The MIC is defined at the lowest antibiotic concentration that completely inhibits the growth of bacteria. However, for obligate intracellular bacteria, such as *O. tsutsugamushi*, which require host cells for multiplication, such conventional AST methods cannot be used. For intracellular pathogens, such as *O. tsutsugamushi*, growth in *in vitro* cell culture can be monitored by methods such as plaque assay,^14–20^ immunofluorescence assay (IFA)^21^ and flow cytometry.^21–23^ However, these methods are time-consuming (∼16–19 days), labour intensive and difficult to interpret.

Quantitative real-time PCR (qPCR) has been used for the early diagnosis and monitoring of drug susceptibility patterns for *O. tsutsugamushi*^24^^,^^25^ and other intracellular bacteria such as *Rickettsia* spp. and *Coxiella burnetii*.^26–29^ However, these studies use a variety of timepoints and multiplicity of infection (moi) ratios making them time-consuming and laborious and not suitable for the routine screening of clinical isolates in endemic settings.

The purpose of this study was to develop a standardized simplified AST method using qPCR and a single antibiotic concentration at a specific timepoint to screen *O. tsutsugamushi* clinical isolates to facilitate large-scale AST.

## Materials and methods

### Standard methods for growth and quantification

#### Cell culture

The mouse fibroblast cell line (L929) was maintained in RPMI 1640 (Gibco, Invitrogen, USA), supplemented with 10% FBS (Gibco, Invitrogen, USA) and incubated at 35°C in a humidified atmosphere with 5% CO_2_, as described previously.^30^

#### Antibiotics

Azithromycin (analytical standard, Sigma–Aldrich, UK), chloramphenicol (BioReagent, suitable for plant culture, Sigma–Aldrich, UK), doxycycline hyclate (>98% purified by HPLC, Sigma–Aldrich, UK) and ofloxacin (fluoroquinolone antibiotic, ≥99% purified by HPLC, Sigma–Aldrich, UK) were used for this study.

#### O. tsutsugamushi reference strains

Eight susceptible or putatively resistant *O. tsutsugamushi* isolates were used (Table 1). These included three strains regarded as antibiotic susceptible: Karp, Kato and Gilliam (obtained from the Australian Rickettsial Reference Laboratory, Geelong, Australia). UT76 was isolated from a patient in Udon Thani, Thailand.^31^ TA763 was isolated from a Rajah rat (*Rattus rajah*) in Ubon Ratchathani, Thailand.^32^ The putatively doxycycline-resistant strains AFC-3, AFSC-4 and AFSC-7 were kindly provided by the Naval Medical Research Center (NMRC, Silver Springs, MD, USA) (Table 1).

**Table 1.** List of *O. tsutsugamushi* reference strains and *in vitro* antibiotic susceptibility reports used in the development of the standardized AST method

Strain	Country of isolation (year)	Doxycycline	Azithromycin	Chloramphenicol	Ofloxacin	Reference(s)
Karp	New Guinea (1943)	S	S	S	NA	^47^^,^^48^
Kato	Japan (1952)	S	NA	S	R	^39^^,^^48^^,^^49^
Gilliam	Burma (1944)	S	NA	S	NA	^48^^,^^50^
UT76	Thailand (2003)	NA	NA	NA	NA	^31^
TA763	Thailand (1963)	NA	NA	NA	NA	^32^
AFC-3	Thailand (1991)	R	NA	S	NA	^9^
AFSC-4	Thailand (1990)	R	S	NA	NA	^37^
AFSC-7	Thailand (1990)	R	NA	NA	NA	^51^

NA, antibiotic susceptibility reports not available; S, susceptible; R, resistant.

#### Ethics statement

This study was approved by the Ethics Committee of the Faculty of Tropical Medicine, Mahidol University, Thailand (approval number MUTM 2016-076-02). All bacteria in this study were isolated from patients who provided written informed consent (and parents or legal guardians of any children provided written informed consent on their behalf).

#### O. tsutsugamushi patient isolates

Fifty-one *O. tsutsugamushi* clinical isolates (Thailand, *n *=* *6; Lao PDR, *n *=* *45) collected from two study sites [Lao-Oxford-Mahosot-Hospital-Wellcome Trust Research Unit (LOMWRU), Vientiane, Lao PDR and Chiangrai Clinical Research Unit (CCRU), Chiangrai, Thailand] were included. These isolates were collected as a part of multiple fever aetiology studies.^5^^,^^33^^,^^34^*O. tsutsugamushi* culture from EDTA blood and subsequent identification and storage was performed as described previously.^30^^,^^35^ The infectivity titre, defined as the DNA-derived bacterial load of the inoculum, was determined using qPCR before freezing (cut-off = 10^8^–10^9^ copies/μL) and before infection of cell lines.^36^

#### Preparation of O. tsutsugamushi inoculum

Prior to every experiment the frozen inoculate was thawed at 37°C, transferred to a 1.5 mL tube and centrifuged at 18 000 **g** for 5 min. Sucrose-phosphate-glutamate (SPG) freezing buffer was discarded and the bacteria resuspended in fresh cell culture media and transferred to 2 mL safe-lock microfuge tubes. The cells were lysed using a vortex mixer vigorously at maximum speed for 1 min. Lysed infected cells were centrifuged at 50 **g** for 3 min to separate host cell debris. The supernatant was transferred into a fresh tube and used in the experiments.

#### Bacterial quantification by qPCR targeting the 47 kDa gene

DNA from infected cells was extracted using the HotShot technique as described previously.^30^ The *O. tsutsugamushi* DNA copy number was measured using qPCR targeting the 47 kDa gene as previously described,^30^^,^^36^ using 1 μL DNA templates. DNA bacterial load under antibiotic treatment was compared with the load with no antibiotic treatment as a control.

### Optimized bacterial growth and harvesting conditions

#### moi validation

To optimize the condition to determine the MIC, *O. tsutsugamushi* strains were inoculated onto monolayers of L929 cells in 24-well plates. Pathogen numbers (DNA copies) corresponded to an moi of 100:1 (*Orientia* DNA 1 × 10^6^ copies per 1 × 10^4^ of L929 cells) or 1000:1 (*Orientia* DNA 1 × 10^7^ copies per 1 × 10^4^ of L929 cells). After incubation for 2 h, the inoculum was removed and infected cells were washed twice with 1× PBS before adding culture media. To identify the optimal moi, the growth curves from the two mois were compared.

#### Cell harvesting method

Cell monolayers were harvested by trypsinization (Trypsin-EDTA, Gibco, 10×, UK) or scraping (cell scraper, Corning, USA). Infected cells were collected every day throughout the 12 days. Pathogens were quantified by qPCR as described above.

#### Growth kinetics of susceptible and resistant O. tsutsugamushi strains

L929 cells were inoculated into 24-well plates and incubated at 35°C overnight. Cell monolayers were then infected with *O. tsutsugamushi* at a final moi of 100:1. Infected cells were cultured for 12 days and harvested at days 0, 3, 4, 5, 6, 7, 10, 11 and 12. Bacterial loads of each day’s harvest were measured by qPCR.

#### Data generation and analysis

To ensure reproducibility of results, all experiments in this study were conducted in three independent experiments and each experiment was conducted in triplicate. The mean and standard deviation (SD) were calculated for individual three experiments. An *F*-test was used to compare variances between the scraping and trypsinization methods and ANOVA was used to compare bacterial growth at the generated MIC in different experiments using STATA v14.2 (College Station, TX, USA). Bacterial copy numbers between days 7 and 10 were compared using Student’s *t*-test. To standardize between experiments the relative growth with different antibiotics was normalized by comparison with non-antibiotic controls. Data visualization and statistical analysis was performed using GraphPad Prism 7.0 (GraphPad Software, Inc., San Diego, CA, USA), unless otherwise stated. Results were considered significantly different if *P *<* *0.05.

### AST method

#### Antibiotics

Two-fold serial dilutions of azithromycin (range from 0.00005 to 0.0625 mg/L), chloramphenicol (range from 0.009 to 10 mg/L), doxycycline (range from 0.0002 to 0.250 mg/L) and ofloxacin (range from 0.5 to 32 mg/L) were used to include concentrations previously reported.^37–39^

#### AST

Infected cells were washed twice with 1× PBS before adding culture media with or without antibiotics. Infected cells were incubated at 35°C with 5% CO_2_ for 10 days, prior to harvest. As the bacterial growth was determined using DNA copy numbers, it is not possible to determine whether all DNA was derived from live bacteria. We determined MICs of azithromycin, chloramphenicol, doxycycline and ofloxacin as the concentrations that inhibit >90% of bacterial growth. As the MIC_90_ was used to calculate the cut-off for screening, any isolates that were able to grow more than 10% when compared with non-antibiotic controls were suspected to be resistant and investigated further with a series of antibiotic dilutions to confirm the MICs and review clinical data for that isolate.

#### Determination of single screening concentration

As described by CLSI, ECOFFinder was used to determine epidemiological cut-off (ECOFF) values.^40^ The ECOFF is determined from the log_2_ estimates of the mean and SD using non-linear regression curve fitting of pooled cumulative datasets from our study (https://clsi.org/education/microbiology/ecoffinder/). An ECOFF of 99% means that 99 out of 100 bacterial isolates are susceptible with the concentration used.^40^ The ECOFF approach was selected to determine the screening concentration, as it is closest to the conventional ‘eyeball’ method used for extracellular organisms.^40^^,^^41^ The ‘eyeball’ method is visual inspection of MIC distributions to determine cut-offs.

## Results

In order to develop a standardized screening method for *O. tsutsugamushi* antimicrobial susceptibility, the conditions for a reproducible method with limited variation between experiments and isolates were defined in the first step.

### Development of standardized growth and harvesting conditions

**Figure 1.**
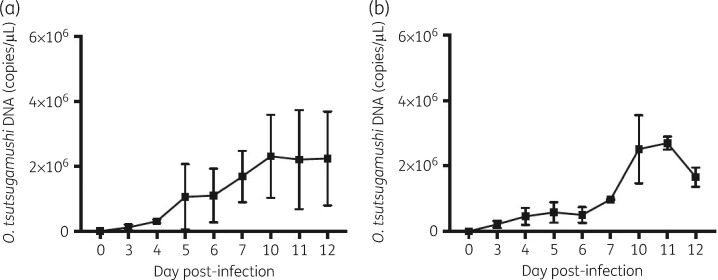
Comparison of variation between replicates of *O. tsutsugamushi* strain Kato growth using different harvesting methods. (a) Scraping method. (b) Trypsinization method.

Variations between trypsinization and the scraping method were observed between different experiments, with the SD of *Orientia* DNA in the experiment being higher when using the scraper compared with trypsinization at day 5 (1 001 275 copies/μL versus 316 190 copies/μL), day 6 (825 686 copies/μL versus 242 163 copies/μL), day 7 (786 957 copies/μL versus 12 590 copies/μL), day 10 (1 275 464 copies/μL versus 197 736 copies/μL), day 11 (1 520 186 copies/μL versus 197 736 copies/μL) and day 12 (1 440 961 copies/μL versus 292 027 copies/μL) (Figure 1). However, none of these pairs was significantly different (*P *>* *0.05).

**Figure 2.**
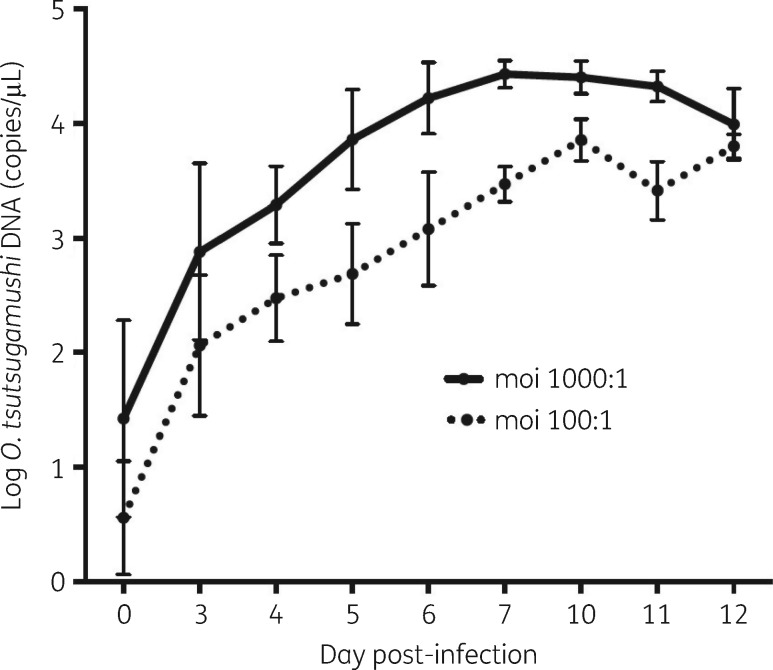
Comparison of different mois of *O. tsutsugamushi* strain Kato*.* Bacterial infection using mois of 1000:1 (continuous line) and 100:1 (broken line) over 12 days with growth peaks at day 7 (moi = 1000:1) and at day 10 (moi = 100:1). The plotted data points represent the mean and SD of three independent experiments.

The growth rates between mois of 1000:1 and 100:1 were similar over 12 days when comparing the growth curve slopes. The slope was calculated from the difference in bacterial load between days divided by the number of days (1000:1 slope = 0.39 versus 100:1 slope = 0.35) (Figure 2).

Seven known susceptible and putatively resistant strains (Karp, Kato, Gilliam, UT76, AFC-3, AFSC-4 and TA763) were used to identify growth peaks. Although there were differences in doubling times between strains, two growth peaks were consistently identified at day 7 and day 10 (Figure S1, available as Supplementary data at *JAC* Online). Six of the seven (85.7%) strains showed no significant differences in bacterial loads on day 7 or day 10. However, bacterial loads on day 10 were significantly higher than day 7 for the Kato strain and therefore day 10 was chosen as the single harvesting day (Table 2). In summary the optimum conditions used for further investigation were as follows: *O. tsutsugamushi* inoculated onto monolayers with an moi of 1:100 in 24-well plates and cell monolayers harvested by trypsinization at day 10.

**Table 2.** Growth data for *O. tsutsugamushi* strains

Strain	Estimated doubling time (h)	Log of bacterial load (95% CI)		*P*
		day 7	day 10	
Karp	15.05	5.193 (4.791–5.595)	5.142 (4.907–5.377)	0.6639
Kato	19.85	5.593 (5.363–5.824)	5.847 (5.732–5.961)	0.0134^a^
Gilliam	12.03	5.537 (4.999–6.077)	5.887 (5.775–5.999)	0.0524
AFC-3	9.50	6.363 (5.650–7.076)	6.182 (5.857–6.508)	0.3782
AFSC-4	10.53	6.221 (6.000–6.442)	6.227 (6.050–6.404)	0.9334
TA763	13.89	5.367 (5.160–5.897)	5.590 (4.876–6.303)	0.6629
UT76	14.42	5.444 (5.354–5.535)	5.935 (5.113–6.756)	0.1223

a Statistically significant (*P *<* *0.05).

### Development of screening methodology

#### AST of susceptible/resistant strains

The optimized growth methods described above were used to determine the MICs of multiple strains (Table 3). The results suggest that the MICs of chloramphenicol for the Karp and AFSC-4 strains are similar (2.5 mg/L) (Table 3). However, the MIC of azithromycin was estimated to be 2-fold lower for Karp than for AFSC-4 (0.0156 mg/L versus 0.0313 mg/L) and the MIC of doxycycline for Karp was 4-fold lower when compared with AFSC-4, which is consistent with published data (Table 3 and Figure S2). The MICs of these two strains and other strains were consistent across three independent experiments (Table 3 and Figure S3). Moreover, the MIC determined of ofloxacin for seven strains of *O. tsutsugamushi* was 8 mg/L, which is the same MIC as in a published report of the Kato strain (Table 3 and Figure S4). This suggests that the methodology developed here gives similar results for MICs compared with published methods.

**Table 3.** MICs of four antibiotics for *O. tsutsugamushi* reference strains

Strain	MIC (mg/L) this study/reference data			
	azithromycin	doxycycline	chloramphenicol	ofloxacin
Kato	0.0039/NA	0.0313/NA	2.500/1.560^52^	8/8^39^
Gilliam	0.0313/NA	0.1250/NA	1.250/0.781^52^	8/NA
AFC-3	0.0156/NA	0.1250/>4^9^	1.250/NA	8/NA
TA763	0.0313/NA	0.0156/NA	0.156/NA	8/NA
UT76	0.0156/NA	0.0625/NA	1.250/NA	8/NA
Karp	0.0156/0.0078^37^	0.0625/0.0625^37^	2.500/1.560^52^	8/NA
AFSC-4	0.0313/0.0156^37^	0.250/>0.250^37^	2.500/NA	8/NA

NA, no comparative data available in the literature.

Superscript numbers correspond to the references for these published data.

#### Determination of single screening concentration

**Figure 3.**
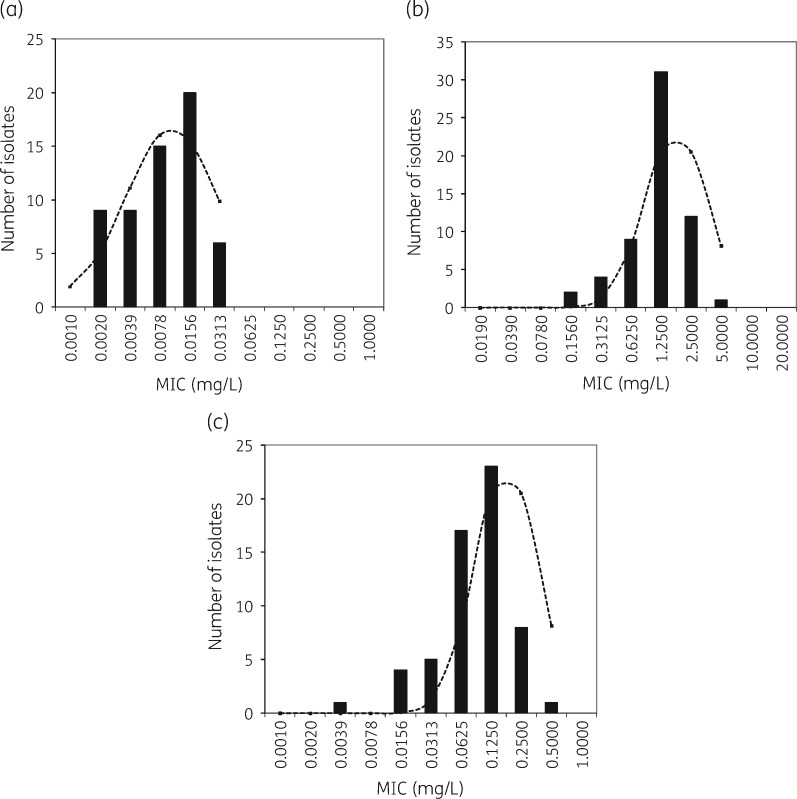
Distribution of MIC values of azithromycin (a), chloramphenicol (b) and doxycycline (c). The dashed line represents the best-fit line of the MIC distribution of susceptible bacterial strain observations calculated by the ECOFFinder.

To determine a single screening concentration to be used for routine AST, the MICs of 59 Lao and Thai patient isolates were determined using the above methods. MICs were further analysed using ECOFFinder analysis to identify the optimal single screening concentration. The modes of azithromycin, chloramphenicol and doxycycline MICs were 0.0156 mg/L (18/59, 30.5%), 1.25 mg/L (31/59, 52.5%) and 0.125 mg/L (24/59, 40.7%), respectively (Figure 3). As all isolates fell within the normal distribution curve calculated by ECOFFinder and no outliers were detected, all tested isolates were considered susceptible to the three antibiotics (Figure 3).

Using ECOFF99%, the corresponding susceptible cut-offs of azithromycin, chloramphenicol and doxycycline were 0.125 mg/L, 8 mg/L and 1 mg/L, respectively.

Therefore, based on optimization and ECOFF, the final simplified AST screening method proposed for *O. tsutsugamushi* is as follows: inoculate with an moi of 100:1 in 24-well plates; incubate with 0.125 mg/L, 8 mg/L and 1 mg/L azithromycin, chloramphenicol and doxycycline, respectively; grow for 10 days; harvest using trypsinization; and determine the pathogen load by qPCR.

## Discussion

This article describes the development of a novel AST screening methodology for *O. tsutsugamushi*, a significant cause of morbidity and mortality in rural Asia.^4^^,^^5^ Firstly, we optimized inoculation, growth and harvesting conditions to establish a reproducible and robust tool that can easily be implemented in multiple biosafety level 3 (BSL-3) laboratories. A single timepoint and single concentration for AST testing was identified to simplify procedures and optimize workload, human capacity and consumable requirements. This method is a significant improvement over previously described tools that also only used one timepoint but required a number of antibiotic concentrations.^28^^,^^29^^,^^42^*O. tsutsugamushi* growth was quantified using qPCR, as this method is more specific, sensitive and faster than flow cytometry, IFA and plaque assay.^14–23^^,^^26^^,^^37^^,^^43^ In comparison with published data derived two decades ago the MICs described here varied by 2-fold,^37^ likely because of the different quantification method as well as the frequency of passages of the *O. tsutsugamushi* stocks. However, the MIC of ofloxacin, which is an ineffective antibiotic for this pathogen,^44^ shows the same value as derived in an earlier study.^39^

The AST methodology described here is able to determine the susceptibility of *O. tsutsugamushi* to the three common antibiotics used for scrub typhus therapy (azithromycin, chloramphenicol and doxycycline) with one, rather than multiple, screening concentrations and yielding results in 10 days. Owing to the complexity of intracellular pathogen growth and BSL-3 requirements this remains a relatively complex process. However, compared with earlier methods that used up to 10 concentrations per antibiotic per isolate, the reduction in consumables including culture plates, cell culture reagents and hands-on time is by at least a factor of eight. Additionally, the described method allows not only simpler data collection but also allows higher-throughput testing. It is possible to screen 48 isolates at the same time for three antibiotics, whereas only 6 isolates at a time could comfortably be tested with previous protocols. Such high-throughput processing would allow more widespread monitoring of this emerging pathogen at national or regional laboratories with BSL-3 facilities to inform treatment guidelines in a timely fashion. Other intracellular pathogens, such as *Rickettsia typhi* and *C. burnetii*, also represent major causes of disease and the current method could be adapted for these organisms.

The single screening concentration was determined using the ECOFF, a previously published method for non-intracellular pathogens, such as *Candida auris*.^45^ This report is the first using this approach for intracellular organisms. The current study compared Karp, a doxycycline-susceptible strain, with two apparently doxycycline-resistant strains (AFSC-4 and AFC-3).^9^^,^^37^ The results are in line with very recent data, which suggest that these strains are not resistant to doxycycline,^3^ in contrast to the data published for these strains 21 years ago.^9^^,^^37^ The data from the current study show that the MICs of both putatively resistant strains are comparable with non-resistant isolates, even if the AFC-3 and AFSC-4 MICs are slightly higher than the Karp strain. These differences over 21 years could have resulted from multiple bacterial passages affecting the antimicrobial susceptibility of *O. tsutsugamushi*. The differences in detection methods between previous studies and our experiments may also have contributed to these differences in MICs.

Limitations of the study include that only isolates from Laos and Thailand were used and that only MIC data were used to calculate the ECOFF while fever clearance time, pharmacological and genetic marker considerations were not included.^40^ Further, conventionally, ECOFF calculation requires 100 isolates representing multiple different laboratories.^46^ However, there are very few laboratories in the world that perform *O. tsutsugamushi* culture from buffy coat. The current requirement for culture at BSL-3 limits the technique to a few specialized centres. With the growing understanding of the importance of this pathogen and the use of modern techniques, cultured isolates should become more available and our method could help to characterize their antimicrobial susceptibility rapidly. One key limitation is that by using DNA as the endpoint parameter for AST determination it is unclear whether the DNA is from live or dead bacteria.

The simultaneous rise of AMR as a global public health problem and the great expansion of the known distribution of scrub typhus has informed the necessity of developing the described methodology. These data, and those of Kelly *et al.*,^3^ question the existence of doxycycline- and chloramphenicol-resistant scrub typhus but give additional support to the evidence that *O. tsutsugamushi* is intrinsically resistant to fluoroquinolones.^39^^,^^44^ However, the wide genetic diversity of *O. tsutsugamushi* suggests the possibility of geographical diversity in antimicrobial susceptibility patterns that should be investigated. The simplified methodology could be used to screen large isolate sets to quantify local *O. tsutsugamushi* susceptibility to key antibiotics and inform treatment guidelines.

## Supplementary Material

Supplementary Data
