# Supplementary material for: Novel high-throughput screening method using quantitative PCR to determine the antimicrobial susceptibility of Orientia tsutsugamushi clinical isolates
Source: J Antimicrob Chemother. 2018 Oct 6;74(1):74–81. doi: 10.1093/jac/dky402 (PMC6293087; doi:10.1093/jac/dky402)
Supplement: Supplementary Data [file dky402_supplementary_data.doc]

**Supplementary data**

**
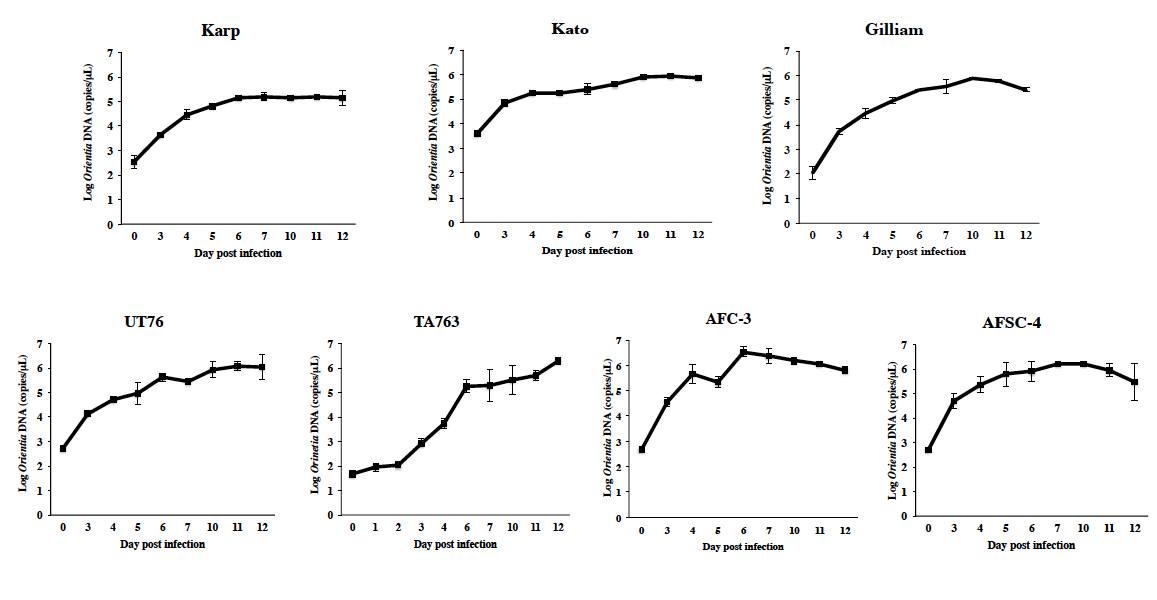
**

**Figure S1.** Growth kinetics and doubling time of susceptible and resistance strains of *O. tsutsugamushi* for 12 days.

**Figure S2.** MICs for Karp and AFSC-4. Determination of MIC of three antibiotics, azithromycin (a, d), doxycycline (b, e) and chloramphenicol (c, f) using percent of bacteria growth between doxycycline susceptible strains (Karp) and doxycycline resistant strains (AFSC-4), black arrow indicates MIC in this study and dash arrow indicates MIC in previous study. P < 0.05 was considered as statistically significantly difference in growth between experiments.

**Figure S3.** Determination of MIC of three antibiotics for *O. tsutsugamushi* in different strains. Azithromycin (a, d, g, j and m), doxycycline (b, e, h, k and n) and chloramphenicol (c, f, i, l and o) using percent of bacteria growth of strain Kato, Gilliam, AFC-3, TA763 and UT76, respectively. Black arrow indicates MIC in this study and dash arrow indicates MIC in previous study. P < 0.05 was considered as statistically significantly difference in growth between experiments.

**Figure S4.** MIC determination of ofloxacin for *O. tsutsugamushi* in seven strains(Karp, Kato, Gilliam, UT76, AFC-3, AFSC-4 and TA763). The black arrow indicates MIC in this study and dashed arrow indicates MIC in a previously published study.^39^ P < 0.05 was considered as statistically significantly difference in growth between experiments.
